# Factors influencing mask-wearing behavior in the context of COVID-19 severity risks in the post-COVID-19 era: a Japanese Nationwide Epidemiological Survey in 2023

**DOI:** 10.1265/ehpm.24-00138

**Published:** 2025-05-27

**Authors:** Shingo Noguchi, Tomohiro Ishimaru, Kazuhiro Yatera, Yoshihisa Fujino, Masayoshi Zaitsu, Takahiro Tabuchi

**Affiliations:** 1 Department of Respiratory Medicine, University of Occupational and Environmental Health, Japan; 2 Department of Respiratory Medicine, Tobata General Hospital; 3 Department of Medical Humanities, School of Medicine, University of Occupational and Environmental Health, Japan; 4 Department of Environmental Epidemiology, Institute of Industrial Ecological Sciences, University of Occupational and Environmental Health, Japan; 5 Center for Research of the Aging Workforce, University of Occupational and Environmental Health, Japan; 6 Division of Epidemiology, School of Public Health, Tohoku University Graduate School of Medicine

**Keywords:** Mask, Risk factors, Severe COVID-19

## Abstract

**Background:**

Although the global COVID-19 mortality rate is decreasing, COVID-19 remains an infectious disease with a high mortality rate, especially in older adults and individuals with comorbidities. In Japan, mask-wearing has been left to individual discretion since March 13, 2023, but remains a key protective measure. This study aimed to identify factors influencing individual mask-wearing behavior in post COVID-19 era, with a focus on risk factors for severe COVID-19.

**Methods:**

Data from 33,000 participants, obtained from the Japan COVID-19 and Society Internet Survey 2023, were used, which was conducted from September 25 to November 17, 2023. Participants were randomly selected from approximately 2.2 million panelists from a nationwide Japanese Internet research company, with sampling adjusted by age, sex, and living area to match the population distribution in Japan. The association between wearing a mask and risk factors for severe COVID-19 (age, sex, smoking, COVID-19 vaccination, history of COVID-19, body mass index (BMI), and comorbid conditions) was evaluated using univariate and multivariate analyses.

**Results:**

In total, 28,481 individuals were included, of whom 18,371 (64.5%) answered that they wore masks. After adjusting for confounders, older age (adjusted relative risk [RR], 1.50; 95% confidence interval [CI], 1.45–1.55 for “75–83” years), no history of COVID-19 (adjusted RR, 1.06; 95% CI, 1.04–1.08), low BMI (adjusted RR, 1.04; 95% CI, 1.02–1.07), and increased number of comorbid conditions (adjusted RR, 1.11; 95% CI, 1.05–1.18 for three or more) were significant positive factors for wearing a mask. In contrast, men (adjusted RR, 0.89; 95% CI, 0.87–0.90), no COVID-19 vaccination (adjusted RR, 0.78; 95% CI, 0.76–0.81), and current smoking history (adjusted RR, 0.96; 95% CI, 0.93–0.99) were significant negative factors.

**Conclusion:**

We demonstrated that mask-wearing behavior differed based on individual risk factors for severe COVID-19, with some risk factors negatively influencing mask use in Japan. It may be necessary to recommend mask-wearing for these individuals, especially during situations such as COVID-19 epidemic season or the onset of epidemics, considering individual mask-wearing behavior.

**Supplementary information:**

The online version contains supplementary material available at https://doi.org/10.1265/ehpm.24-00138.

## Background

Severe acute respiratory syndrome coronavirus 2 (SARS-CoV-2), which causes coronavirus disease 2019 (COVID-19), has short- and long-range transmission modes, such as direct or indirect contact, droplets, and aerosols with increased transmissibility [1, 2], and this virus particularly exists in saliva at high concentrations [3]. Wearing a mask is effective in reducing the infection and fatalities of COVID-19, and it has been known to play an important role in COVID-19 pandemic [4]; for example, it has been reported to reduce the incidence of COVID-19 by a relative risk of 0.47 (95% confidence interval [CI], 0.29–0.75) in systematic reviews, although heterogeneity was substantial [4–8]. In addition, COVID-19 has the characteristic of being transmitted before the onset of symptoms; therefore, ordinary mask-wearing behavior also plays a role in reducing transmission of COVID-19 [9, 10].

Many countries have recommended mask-wearing behavior as an important prevention measure since the beginning of the COVID-19 pandemic and have introduced mask mandates, especially in crowded spaces such as public indoor spaces, public transport, hospitals, and other healthcare settings [11]. However, owing to the decreased rate of severity, hospitalization, and mortality caused by COVID-19, many countries have gradually revised regulations regarding the mandatory wearing of masks, and people are no longer required to wear masks in public. Even in Japan, strict infectious control measures, including mask-wearing behavior for COVID-19 prevention, have been implemented since the beginning of the COVID-19 pandemic. Although the Japanese government never mandated mask-wearing, it recommended their use throughout the COVID-19 pandemic, except outdoors or when physical distancing can be maintained or conversation is minimal from May 2022. Japan has consistently demonstrated high mask usage so far [12]. However, various infectious control measures have been released, and mask-wearing behavior has also been left to the discretion of the individual since March 13, 2023 [13].

Declined death rates of patients with COVID-19 were observed because of changes in SARS-CoV-2 variants, partly due to increased immunity levels and improved clinical care [14]; however, COVID-19 remains an infectious disease with a high mortality rate for older adults and individuals with comorbidities [15, 16]. Moreover, it deteriorates physical activity in older individuals [17, 18]. Various risk factors are associated with the severity of COVID-19. For example, older age is significantly associated with high mortality risk [14, 15], and an increased number of comorbid conditions also leads to an increased risk ratio for death in patients with COVID-19; 2.6 times higher risk ratio for death is reported in patients with 2–5 comorbid conditions than in patients with no comorbid conditions [19]. In addition to the above factors, being unvaccinated and not having a history of COVID-19 are high-risk factors for death due to COVID-19 [14]. COVID-19 vaccination has become widespread worldwide; however, vaccination alone is not enough to control the infection rate, death, and transmission of SARS-CoV-2 [20] because the efficacies of vaccination for COVID-19 are influenced by various factors, such as age, sex, type of vaccine, elapsed time after vaccination, and viral mutation. Therefore, personal prevention measures, including wearing a mask, remain an important method to protect oneself and/or others from the risks of infection and death due to COVID-19, even if wearing a mask is based on individual discretion. In addition, maintaining to wear a mask can not only be cost-effective but also cost-saving [20, 21].

In Japan, over 16,000 deaths from COVID-19 have been reported from May to November 2023 since restrictions were eased [22], and the reduction of mask use could result in additional deaths, as estimated using serial cross-sectional data from Tokyo, Japan [23]. Therefore, COVID-19 remains a disease that requires ongoing attention, especially among groups with risk factors for severe COVID-19, and mask-wearing continues to play a key role in COVID-19 prevention. However, it is unclear whether people have stopped wearing masks in response to changes in policy or continue to wear a mask for health and hygiene reasons after the revision of regulations.

Therefore, this study aimed to identify which risk factors for severe COVID-19 influence mask-wearing behaviors in the post-COVID-19 era, focusing on the risk factors for severe COVID-19.

## Methods

### Data source

We used data from the Japan COVID-19 and Society Internet Survey (JACSIS) 2023, an ongoing cross-sectional Internet cohort study designed to solve various social and health problems related to COVID-19 in Japan. The JACSIS survey was conducted by Rakuten Insight Corporation, Tokyo, Japan, which is a major nationwide internet research agency in Japan. Participants were selected by simple random sampling using a computer algorism from Rakuten Insight databases, which had approximately 2.2 million panelists in 2019 [24]. This sampling was adjusted by age, sex, and living area (prefecture) in order to match the population distribution in Japan [25]. The JACSIS 2023 survey was conducted from September 25, 2023, to November 17, 2023. The 26,872 out of 46,840 participants (response rate, 57.4%) were recruited from the follow-up survey between 2015 and 2023 were firstly included, and 6,128 participants aged 16–79 were newly included from panel members [26]. This study was approved by the Research Ethics Committee of Osaka International Cancer Institute (No. 20084). This study was conducted in accordance with the principles of the Declaration of Helsinki. Web-based informed consent was obtained from all participants via electromagnetic means upon registration.

### Inclusion and exclusion criteria

Data from all respondents (33,000) of the JACSIS 2023 survey were included. The raw data didn’t have missing one because the survey design ensured that all survey questions must be answered [25]. However, inconsistent responses were possible, and the respondents with any invalid responses were defined as follows and excluded from this study to ensure the validity of this survey: not selecting the correct answer for the question “Please select the second from the bottom out of five options,” selecting the use of all drugs for the question “Do you currently use any drug out of eight items?,” selecting all items for the question “Do you currently have any chronic illness?,” reporting the total number of people living together as >15 persons, and answering in a short response time of <15 min.

### Risk factors for severe COVID-19

Data concerning age, sex, smoking status, COVID-19 vaccination, history of COVID-19, body mass index (BMI), and nine comorbid conditions (cancer, cerebrovascular disease, cardiovascular disease, chronic lung disease, chronic liver disease, chronic kidney disease, diabetes, immunosuppressed status, and hypertension), which are commonly identified as significant contributors to severe COVID-19 outcome, were extracted as risk factors for severe COVID-19 [16, 19, 27]. The age and smoking status were divided into five (16–39, 40–49, 50–64, 65–74, and 75–83 years) and three (“current,” “past,” “never”) groups, respectively. The history of COVID-19 vaccination was classified into three groups (none, one or two time(s), and three or more times), and the history of COVID-19 was defined as having SARS-CoV-2 infection at least once. In addition, comorbid conditions were reclassified into four groups (0, 1, 2, and ≥3) according to the number of comorbid conditions described above.

### Definition of wearing a mask

The following question was asked, “Do you basically wear a mask in ordinary life except when wearing a mask is recommended (such as in medical institutions, care facilities for older adults, crowded trains or buses, and when required by your workplace)?” and participants were defined as wearing a mask when they answered “Yes.” In addition, the reason for wearing a mask was determined using 11 items, targeting participants who answered that they wore a mask.

### Other variables

The variables included annual household income (<2,000,000 yen, 2,000,000–3,999,999 yen, 4,000,000–6,999,999 yen, and ≥7,000,000 yen, unknown), education (junior high or high school, vocational school or college, and university or graduate school), occupation (self-employed, part-time/full-time employee, manager or executive, and unemployed), and marital status (married, divorced or widowed, and single).

### Statistical analysis

Descriptive statistics were used to summarize the demographic characteristics of the study participants, with the number and percentage of participants tabulated according to their mask-wearing behavior (yes or no). Furthermore, a descriptive analysis was conducted to examine the reasons for mask-wearing behavior, and Poisson regression analyses with a robust variance estimator were conducted to identify the factors associated with wearing a mask. In the multivariate analyses, we defined the causal pathway [see Supplementary Fig. 1 in Additional file 1], where mask-wearing status was treated as the outcome, comorbid conditions as the primary exposure, and COVID-19 vaccination status and history of COVID-19 infection as mediators. These factors were analyzed in models adjusted for age, sex, annual household income, education level, occupation, marital status, BMI, and smoking status. In addition, a fully adjusted model was constructed to examine the independent associations of these demographic and lifestyle factors with mask-wearing behavior. Relative risks (RRs) and 95% CIs were calculated to assess the strength and direction of associations. A subgroup analysis was performed to assess the relationship between mask-wearing status and each comorbid condition. All statistical analyses were performed using Stata/SE 16.1 (StataCorp, College Station, TX, USA), with the significance level set at p < 0.05.

## Results

### Baseline characteristics

Among 33,000 participants, 4,519 were excluded owing to invalid responses, and 28,481 were finally included in this study. The general characteristics of the participants are summarized in Table 1. Among the 28,481 enrolled participants, 6,828 (24.0%) were older adults aged ≥65 years, and 14,410 (50.6%) were women. Participants with COVID-19 vaccination three or more times were 21,243 (74.6%), and 8,718 (30.6%) participants had a history of COVID-19. Moreover, 22,323 (78.4%) participants did not have any comorbid conditions, 561 (2.0%) had three or more comorbid conditions, and 18,371 (64.5%) answered that they wore masks.

**Table 1:** General characteristics of participants

	**Total**	**Wearing a mask**	

		**Yes**	**No**
	**(n = 28,481)**	**(n = 18,371)**	**(n = 10,110)**
Age			
16–39 years	10,505 (36.9)	5,656 (30.8)	4,849 (48.0)
40–49 years	4,513 (15.8)	2,855 (15.5)	1,658 (16.4)
50–64 years	6,635 (23.3)	4,611 (25.1)	2,024 (20.0)
65–74 years	4,641 (16.3)	3,514 (19.1)	1,127 (11.1)
75–83 years	2,187 (7.7)	1,735 (9.4)	452 (4.5)
Sex			
Women	14,410 (50.6)	10,043 (54.7)	4,367 (43.2)
Men	14,071 (49.4)	8,328 (45.3)	5,743 (56.8)
Annual household income			
<2,000,000 yen	2,459 (8.6)	1,684 (9.2)	775 (7.7)
2,000,000–3,999,999 yen	5,619 (19.7)	3,904 (21.3)	1,715 (17.0)
4,000,000–6,999,999 yen	7,066 (24.8)	4,421 (24.1)	2,645 (26.2)
≥7,000,000 yen	6,761 (23.7)	3,804 (20.7)	2,957 (29.2)
Unknown	6,576 (23.1)	4,558 (24.8)	2,018 (20.0)
Education			
Junior high or high school	8,025 (28.2)	5,604 (30.5)	2,421 (23.9)
Vocational school or college	5,922 (20.8)	4,146 (22.6)	1,776 (17.6)
University or graduate school	14,534 (51.0)	8,621 (46.9)	5,913 (58.5)
Occupation			
Self-employed	1,899 (6.7)	1,197 (6.5)	702 (6.9)
Part-time employee	4,833 (17.0)	3,414 (18.6)	1,419 (14.0)
Full-time employee	8,614 (30.2)	4,917 (26.8)	3,697 (36.6)
Manager or executive	2,965 (10.4)	1,685 (9.2)	1,280 (12.7)
Unemployed	10,170 (35.7)	7,158 (39.0)	3,012 (29.8)
Marital status			
Married	16,014 (56.2)	10,533 (57.3)	5,481 (54.2)
Divorced or widowed	2,619 (9.2)	1,870 (10.2)	749 (7.4)
Single	9,848 (34.6)	5,968 (32.5)	3,880 (38.4)
COVID-19 vaccination			
3 or more times	21,243 (74.6)	14,522 (79.0)	6,721 (66.5)
1 or 2 time(s)	3,479 (12.2)	1,908 (10.4)	1,571 (15.5)
None	3,759 (13.2)	1,941 (10.6)	1,818 (18.0)
History of COVID-19			
Yes	8,718 (30.6)	5,080 (27.7)	3,638 (36.0)
No	19,763 (69.4)	13,291 (72.3)	6,472 (64.0)
Body mass index			
<18.5 kg/m^2^	3,961 (13.9)	2,644 (14.4)	1,317 (13.0)
18.5–24.9 kg/m^2^	19,628 (68.9)	12,576 (68.5)	7,052 (69.8)
25.0–29.9 kg/m^2^	3,968 (13.9)	2,572 (14.0)	1,396 (13.8)
≥30.0 kg/m^2^	924 (3.2)	579 (3.2)	345 (3.4)
Smoking			
Never	19,292 (67.7)	12,482 (67.9)	6,810 (67.4)
Past	6,098 (21.4)	3,978 (21.7)	2,120 (21.0)
Current	3,091 (10.9)	1,911 (10.4)	1,180 (11.7)
Comorbid conditions			
No conditions	22,323 (78.4)	13,953 (76.0)	8,370 (82.8)
1 condition	4,280 (15.0)	3,095 (16.8)	1,185 (11.7)
2 conditions	1,317 (4.6)	945 (5.1)	372 (3.7)
≥3 conditions	561 (2.0)	378 (2.1)	183 (1.8)
Comorbidities (multiple answers allowed)			
Cancer	608 (2.1)	440 (2.4)	168 (1.7)
Cerebrovascular disease	357 (1.3)	223 (1.2)	134 (1.3)
Cardiovascular disease	568 (2.0)	397 (2.2)	171 (1.7)
Chronic lung disease	262 (0.9)	170 (0.9)	92 (0.9)
Chronic liver disease	302 (1.1)	197 (1.1)	105 (1.0)
Chronic kidney disease	457 (1.6)	312 (1.7)	145 (1.4)
Diabetes	1,689 (5.9)	1,184 (6.4)	505 (5.0)
Immunosuppressed state	544 (1.9)	393 (2.1)	151 (1.5)
Hypertension	4,577 (16.1)	3,275 (17.8)	1,302 (12.9)

COVID-19; coronavirus disease 2019.

The values in parentheses represent the percentage of each subgroup in the total population (n = 28,481), ‘Yes’ (n = 18,371), and ‘No’ (n = 10,110) mask-wearing groups, respectively.

### Reasons for mask-wearing behavior

The reasons for wearing a mask for 18,371 participants are shown in Table 2. The most common reason was “preventing the contraction of respiratory infections, including COVID-19”, followed by “preventing the transmission of respiratory infections to others,” and “health-related reasons beyond infection control, such as allergy prevention.”

**Table 2:** Reasons for mask-wearing behavior (multiple answers allowed)

	**n = 18,371 (%)**
Preventing the contraction of respiratory infections	12,215 (70.3)
Preventing the transmission of respiratory infections to others	10,053 (35.3)
Health-related reasons beyond infection control, such as allergy prevention	3,923 (21.4)
Personal advantages of wearing a mask, such as anonymity or concealment of facial expressions	3,267 (17.8)
Conformity to social norms owing to observed mask-wearing by peers	2,365 (12.9)
Compliance with mask-wearing mandates in professional or educational settings, extending to personal life	2,058 (11.2)
Preference for maintaining mask-wearing habits developed during the pandemic	1,692 (9.2)
Non-specific habitual mask-wearing	1,543 (8.4)
Avoidance of criticism for not wearing a mask	976 (5.3)
Perception of mask non-wearers as inconsiderate	744 (4.0)
Others	378 (2.1)

### Factors associated with mask-wearing behavior

Table 3 shows the univariate and multivariate adjusted RRs for the association between mask-wearing behavior and risk factors for severe COVID-19. After adjusting for confounders, the adjusted RRs for wearing a mask significantly increased in the groups with older individuals compared with that in the group aged “16–39” years; the adjusted RRs (95% CIs) were 1.20 (1.16–1.23), 1.32 (1.28–1.35), 1.43 (1.39–1.47), and 1.50 (1.45–1.55) in individuals aged “40–49,” “50–64,” “65–74,” and “75–83” years, respectively. Compared with women, men showed a significant decrease in wearing a mask (adjusted RR, 0.89; 95% CI, 0.87–0.90). The decreased number of COVID-19 vaccinations was significantly associated with the decrease in wearing a mask, and the adjusted RR (95% CI) for wearing a mask in the individuals with no COVID-19 vaccination was 0.78 (95% CI, 0.76–0.81). In contrast, a significant increase in wearing a mask was observed in those with no history of COVID-19 (adjusted RR, 1.06; 95% CI, 1.04–1.08). BMI ≥ 30.0 kg/m^2^ (adjusted RR, 0.99; 95% CI, 0.95–1.05) was not associated with wearing a mask, but BMI < 18.5 kg/m^2^ (adjusted RR, 1.04; 95% CI, 1.02–1.07) was significantly associated with an increase in wearing a mask. Current smoking history (adjusted RR, 0.96; 95% CI, 0.93–0.99) was significantly related to a decrease in wearing a mask. The individuals with three or more comorbid conditions were significantly associated with wearing a mask than those with no comorbid conditions (adjusted RR, 1.11; 95% CI, 1.05–1.18). Regarding the relationship between wearing a mask and each comorbidity, cancer (adjusted RR, 1.08; 95% CI, 1.03–1.13), cardiovascular disease (adjusted RR, 1.08; 95% CI, 1.02–1.14), chronic lung disease (adjusted RR, 1.10; 95% CI, 1.01–1.20), chronic liver disease (adjusted RR, 1.09; 95% CI, 1.00–1.18), chronic kidney disease (adjusted RR, 1.09; 95% CI, 1.02–1.16), immunosuppressed status (adjusted RR, 1.11; 95% CI, 1.06–1.17), and hypertension (adjusted RR, 1.03; 95% CI, 1.00–1.05) were associated with a significant increase in wearing a mask [see Supplementary Table 1 in Additional file 2].

**Table 3:** Association between mask-wearing behavior and risk factors for severe COVID-19

	**Rate (%)**	**Univariate**			Multivariate*		
	
		**RR**	**95% CI**	**p-value**	**RR**	**95% CI**	**p-value**
Age							
16–39 years	53.8	1	-	-	1	-	-
40–49 years	63.3	1.18	1.14–1.21	<0.001	1.20	1.16–1.23	<0.001
50–64 years	69.5	1.29	1.26–1.32	<0.001	1.32	1.28–1.35	<0.001
65–74 years	75.7	1.41	1.37–1.44	<0.001	1.43	1.39–1.47	<0.001
75–83 years	79.3	1.47	1.43–1.52	<0.001	1.50	1.45–1.55	<0.001
Sex							
Women	69.7	1	-	-	1	-	-
Men	59.2	0.85	0.84–0.86	<0.001	0.89	0.87–0.90	<0.001
COVID-19 vaccination							
3 times or more	68.4	1	-	-	1	-	-
1 or 2 time(s)	54.8	0.80	0.78–0.83	<0.001	0.88	0.85–0.90	<0.001
None	51.6	0.76	0.73–0.78	<0.001	0.78	0.76–0.81	<0.001
History of COVID-19							
Yes	58.3	1	-	-	1	-	-
No	67.3	1.15	1.13–1.18	<0.001	1.06	1.04–1.08	<0.001
Body mass index							
<18.5 kg/m^2^	64.1	1.04	1.02–1.07	0.001	1.04	1.02–1.07	0.002
18.5–24.9 kg/m^2^	66.8	1	-	-	1	-	-
25.0–29.9 kg/m^2^	64.8	1.01	0.99–1.04	0.367	1.01	0.98–1.03	0.629
≥30.0 kg/m^2^	62.7	0.98	0.93–1.03	0.391	0.99	0.95–1.05	0.818
Smoking							
Never	64.7	1	-	-	1	-	-
Past	65.2	1.01	0.99–1.03	0.445	0.98	0.96–1.00	0.069
Current	61.8	0.96	0.93–0.98	0.003	0.96	0.93–0.99	0.007
Comorbid conditions							
No conditions	62.5	1	-	-	1	-	-
1 condition	72.3	1.16	1.13–1.18	0.012	1.04	1.01–1.06	0.002
2 conditions	71.8	1.15	1.11–1.19	<0.001	1.04	1.00–1.08	0.043
≥3 conditions	67.4	1.08	1.02–1.14	0.012	1.11	1.05–1.18	<0.001

RR, relative risk; CI, confidence interval; COVID-19; coronavirus disease 2019.

*Adjusted for age, sex, annual household income, education, occupation, marital status, body mass index, and smoking.

## Discussion

In this study, we investigated the association between mask-wearing behavior and risk factors for severe COVID-19, and found that older age, no history of COVID-19, low BMI, and an increase in the number of comorbid conditions were significantly positively associated factors for the increase in wearing a mask, whereas male sex, no COVID-19 vaccination, and current smoking were significant negative factors.

Among the risk factors for severe COVID-19, older age showed a strong association with mask-wearing behavior, and the increased adjusted RR for wearing a mask was consistent with the increase in the mortality risk of COVID-19 in each age group. Notably, age demonstrated the strongest association with positive mask-wearing behavior among all examined factors [see Supplementary Table 2 in Additional file 2]. The Centers for Disease Control and Prevention showed that the mortality relative risk of COVID-19 was 2.2, 4.3, 6.7, and 8.5 in the 40–49, 50–64, 65–74, and 75–84 years age groups, respectively, when that of the 18–39 years age group was set as the reference [15]. Older age has been reported to be a positive predictor of mask-wearing behavior in previous reports from some countries [28–30], but a recent Japanese report did not find significant differences in mask-wearing behavior according to age group [31]. There may be a noticeable change in the proportion of wearing a mask depending on age after self-discretion in mask-wearing behavior because the ratio of individuals wearing a mask in Japan seems to be high in all age groups (excluding infants) so far. Previous reports have shown a significantly higher ratio of females wearing masks than males [28, 30, 31], and our results were similar to those of previous reports. Women may tend to wear a mask to save makeup [32] and to protect themselves because they often provide the majority of caregiving at home [28], compared to men. Although detailed analysis was not conducted, “Personal advantages of wearing a mask” accounted for 17.8% of the reasons for wearing a mask in this study (Table 2).

Previous reports showed that a history of COVID-19 was associated with low mask usage, whereas COVID-19 vaccination was an independent predictor of high mask usage [33, 34]. In this study, a history of COVID-19 was a significant factor in the decrease in mask-wearing. Generally, a previous history of COVID-19 has a protective effect against the upcoming SARS-CoV-2 infection, although the effect is no longer sufficient in various situations, such as the period of pre-infection and current SARS-CoV-2 viral variants [35–37]. People with a history of COVID-19 may have a reduced fear of COVID-19, which in turn may lead to a decrease in mask use. Additionally, there may be a relationship between mask-wearing behavior and the severity of previous infection with COVID-19; for example, people with mild symptoms of COVID-19 may think that wearing a mask is unnecessary. However, SARS-CoV-2 reinfection increases the rates of hospitalization, mortality, and sequelae [38]. Therefore, infectious control measures due to prevention of SARS-CoV-2 reinfection should be considered, even in individuals with a history of COVID-19. High risks of severity and/or mortality in persons with no history of vaccination are known [39, 40]; however, our results showed a significantly lower rate of wearing a mask in this population than in vaccinated individuals. People with no vaccination history might have had a slight fear of COVID-19 since the beginning of the COVID-19 pandemic and have low awareness of health itself, not limited to COVID-19; however, it is important to note that some people cannot be vaccinated even if they want to, such as those with an allergy.

Regarding the association between mask-wearing behavior and smoking history, current smoking was a significant factor in the decrease in wearing a mask. People with a smoking history may also have a low awareness of health itself. In addition, a report from Hong Kong showed that 81.6% of smokers put a mask under their chin when smoking, and 32.4% did not wear a mask immediately after smoking [41], and they might feel that wearing a mask is a nuisance. Our results showed no significant association between wearing a mask and obesity, similar to a previous report [42]. However, there was a significant positive association between wearing a mask and low BMI. A low BMI is reported to be a poor prognostic factor for respiratory infectious diseases [43]; therefore, individuals with a low BMI may tend to wear a mask.

A previous report showed that hypertension was not a significant factor for mask-wearing behavior in the relationship between mask-wearing behavior and comorbid conditions [42]; however, there are few reports on mask-wearing behavior and comorbid conditions. In the present study, the number of comorbidities was significantly associated with a high rate of mask use. Persons with many comorbidities may fear severe or fatal COVID-19 or may be advised by medical staff to continue wearing a mask during regular hospital/clinic visits. The presence of specific comorbid conditions except cerebrovascular disease and diabetes was a positive predictor of an increase in wearing a mask, suggesting that individuals with specific comorbid conditions are more likely to wear a mask, not only owing to the number of comorbidities but also to the presence of each specific condition. However, the severity of each comorbidity was unavailable in this study, and mask-wearing behavior may have changed according to the severity of each comorbidity. Therefore, further investigation may be needed about these relationships.

Thus, mask-wearing behavior differed according to the differences in risk factors for severe COVID-19. The results of this study were adjusted for demographic factors such as age, sex, education level, and occupation, which have been previously reported to be associated with mask-wearing behavior [44–46]. However, various factors such as personal protective equipment, daily personal infection control measures (other than mask-wearing behavior), the feeling of anxiety and fear for COVID-19, self-identity, political ideology, and trust in governments also influence mask-wearing behavior [45–47]. Although further investigation is needed to clarify the relationship between these factors in post COVID-19 era, there have been no analyses examining the association between individual risk factors for severe COVID-19 and mask-wearing behavior to date.

Our previous data using a large-scale Internet survey, utilizing the same panel data as the data in this present study, showed that the proportion of people wearing a mask was >80% since the end of 2020, and this proportion was 91.2% (26,407 out of 29,963 participants) in a study conducted on February 1–28, 2022 [48]; however, the proportion of people wearing a mask decreased to 65.4% in this study. We cannot clearly explain the relationship between actual mask-wearing behavior and the reasons for continuing to wear masks because we focused on investigating the association of risk factors for severe COVID-19 and mask-wearing behavior in this study. Generally, most barriers to wearing a mask include discomfort and inconvenience [29], and the most common psychological reason for mask-wearing behavior was compliance with others’ mask-wearing norms [31, 42]. However, many of the people wearing masks continue to wear masks to prevent infection and/or avoid transmission of COVID-19 to others, as shown in Table 2.

This study had some limitations. First, this study was self-reported as obtained from questionnaires, and the number of each item, including mask-wearing, might have been overestimated or underestimated and introduce potential reporting bias; however, incorrect answers were omitted to improve the certainty of the answers and responders with discrepancies or inconsistencies were excluded. Moreover, the frequency of mask-wearing may influence our results, although we could not determine the actual frequency of mask-wearing in the responders who answered “Yes” for ordinary mask-wearing behavior. Second, although we conducted statistical adjustment to account for biases, an Internet survey may lead to participant bias, as Internet access or Internet literacy was required and might not fully reflect the general population [49]. In addition, the factors that make people more likely to wear a mask, such as meteorological conditions and specific occupations such as healthcare workers, may not have been considered and may have influenced the results. Third, many participants might get various information from the internet because this study was an online survey. The information obtained from the internet tends to display information that is convenient for them, and their tendency may be different from the information obtained from other media.

## Conclusion

Our findings demonstrate that mask-wearing behavior differs among people with risk factors for severe COVID-19 in Japan, with some risk factors significantly negatively influencing mask-wearing. Therefore, we may need to recommend mask-wearing behavior for these individuals, especially during situations such as COVID-19 epidemic season or onset, although wearing a mask or not has become a matter of personal choice. Thereby, we believe these findings provide imperative information when considering individual mask-wearing behavior in the post-COVID-19 era.

## Abbreviations

BMI: body mass index
CI: confidence interval
COVID-19: coronavirus disease 2019
JACSIS: Japan COVID-19 and Society Internet Survey
RR: relative risk
SARS-CoV-2: severe acute respiratory syndrome coronavirus 2

## Supplementary information

**Additional file 1:** **Supplementary Figure 1.** Causal pathway between each factor and mask-wearing behavior.

**Additional file 2:** **Supplementary Table 1.** Association between mask-wearing behavior and comorbid conditions. **Supplementary Table 2.** Association between mask-wearing behavior and risk factors for severe COVID-19 with standardized coefficients (β).

## Declarations

### Availability of data and material

The datasets used in the current study are available upon reasonable request.
