# Supplementary figures and images for: Factors influencing mask-wearing behavior in the context of COVID-19 severity risks in the post-COVID-19 era: a Japanese Nationwide Epidemiological Survey in 2023
Source: Environ Health Prev Med. 2025 May 27;30:41. doi: 10.1265/ehpm.24-00138 (PMC12127079; doi:10.1265/ehpm.24-00138)

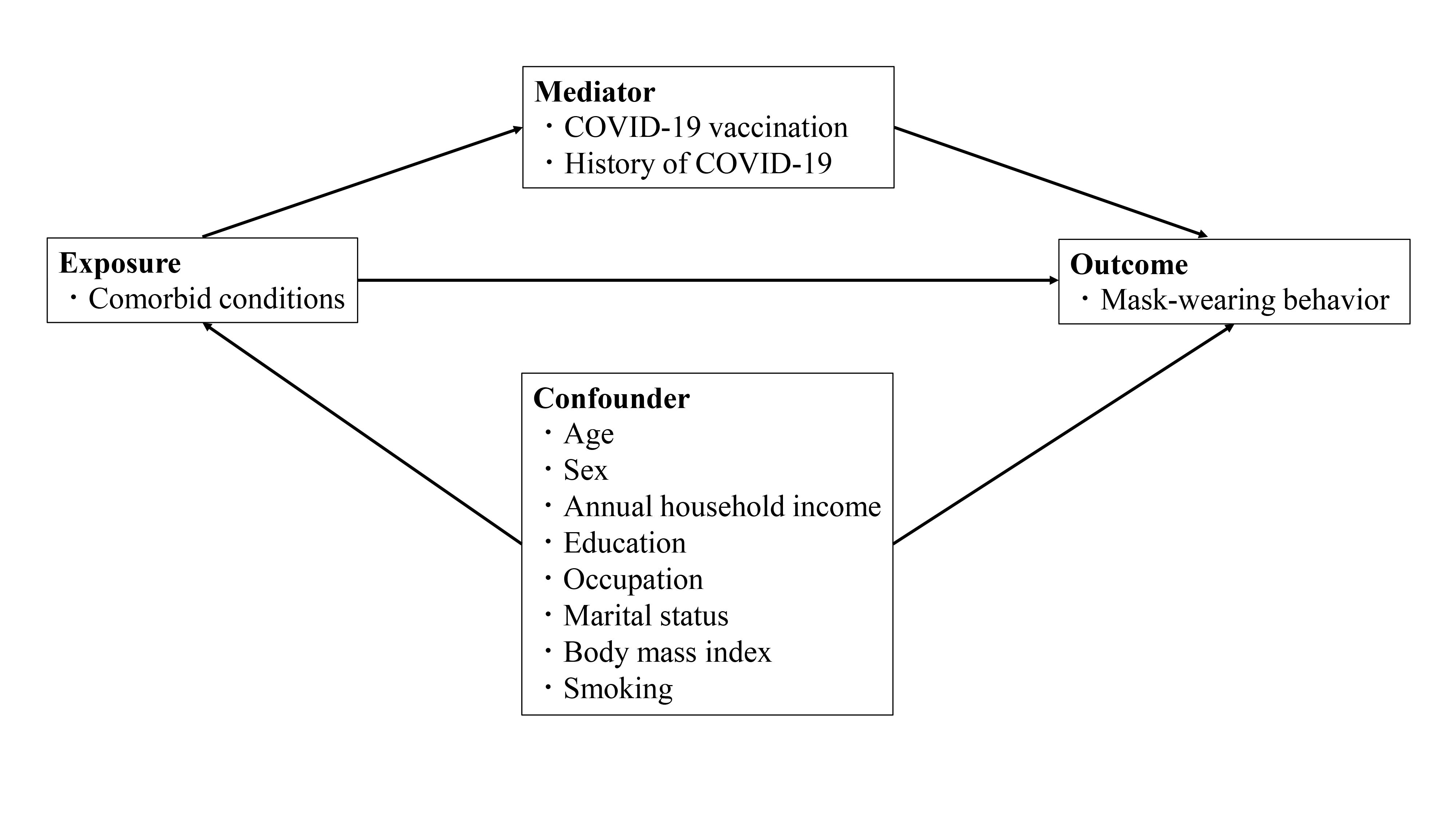

Supplement: Supplementary file 1 — Additional file 1: Supplementary Figure 1. Causal pathway between each factor and mask-wearing behavior. [file ehpm-30-041-s001.tif]
