# Supplementary material for: Factors influencing mask-wearing behavior in the context of COVID-19 severity risks in the post-COVID-19 era: a Japanese Nationwide Epidemiological Survey in 2023
Source: Environ Health Prev Med. 2025 May 27;30:41. doi: 10.1265/ehpm.24-00138 (PMC12127079; doi:10.1265/ehpm.24-00138)
Supplement: Supplementary file 2 — Additional file 2: Supplementary Table 1. Association between mask-wearing behavior and comorbid conditions. Supplementary Table 2. Association between mask-wearing behavior and risk factors for severe COVID-19 with standardized coefficients (β). [file ehpm-30-041-s002.docx]

Supplementary Table 1. Association between mask-wearing behavior and comorbid conditions

|  |  | Rate (%) |  | Univariate | | |  | Multivariate* | | |
| --- | --- | --- | --- | --- | --- | --- | --- | --- | --- | --- |
|  |  |  |  | RR | 95% CI | p-value |  | RR | 95% CI | p-value |
| Cancer | | | | | | | | | | |
|  | No | 64.3 |  | 1 | - | - |  | 1 | - | - |
|  | Yes | 72.4 |  | 1.13 | 1.07–1.18 | < 0.001 |  | 1.08 | 1.03–1.13 | 0.002 |
| Cerebrovascular disease | | | | | | | | | | |
|  | No | 64.5 |  | 1 | - | - |  | 1 | - | - |
|  | Yes | 62.5 |  | 0.97 | 0.89–1.05 | 0.431 |  | 1.02 | 0.94–1.10 | 0.628 |
| Cardiovascular disease | | | | | | | | | | |
|  | No | 64.4 |  | 1 | - | - |  | 1 | - | - |
|  | Yes | 69.9 |  | 1.09 | 1.03–1.15 | 0.003 |  | 1.08 | 1.02–1.14 | 0.005 |
| Chronic lung disease | | | | | | | | | | |
|  | No | 64.5 |  | 1 | - | - |  | 1 | - | - |
|  | Yes | 64.9 |  | 1.01 | 0.92–1.10 | 0.896 |  | 1.10 | 1.01–1.20 | 0.037 |
| Chronic liver disease | | | | | | | | | | |
|  | No | 64.5 |  | 1 | - | - |  | 1 | - | - |
|  | Yes | 65.2 |  | 1.01 | 0.93–1.10 | 0.788 |  | 1.09 | 1.00–1.18 | 0.047 |
| Chronic kidney disease | | | | | | | | | | |
|  | No | 64.4 |  | 1 | - | - |  | 1 | - | - |
|  | Yes | 68.3 |  | 1.06 | 0.99–1.13 | 0.073 |  | 1.09 | 1.02–1.16 | 0.008 |
| Diabetes | | | | | | | | | | |
|  | No | 64.1 |  | 1 | - | - |  | 1 | - | - |
|  | Yes | 70.1 |  | 1.09 | 1.06–1.13 | < 0.001 |  | 1.03 | 0.99–1.07 | 0.054 |
| Immunosuppressed state | | | | | | | | | | |
|  | No | 64.4 |  | 1 | - | - |  | 1 | - | - |
|  | Yes | 72.2 |  | 1.12 | 1.07–1.18 | < 0.001 |  | 1.11 | 1.06–1.17 | <0.001 |
| Hypertension | | | | | | | | | | |
|  | No | 63.2 |  | 1 | - | - |  | 1 | - | - |
|  | Yes | 71.6 |  | 1.13 | 1.11–1.16 | < 0.001 |  | 1.03 | 1.00–1.05 | 0.031 |

RR, relative risk; CI, confidence interval.

*Adjusted for age, sex, annual household income, education, occupation, marital status, body mass index, and smoking.

Supplementary Table 2. Association between mask-wearing behavior and risk factors for severe COVID-19 with standardized coefficients (β)

|  |  | Rate (%) |  | Univariate | | | |  | Multivariate* | | | |
| --- | --- | --- | --- | --- | --- | --- | --- | --- | --- | --- | --- | --- |
|  |  |  |  | β | RR | 95% CI | p-value |  | β | RR | 95% CI | p-value |
| Age | | | | | | | | | | | | |
|  | 16–39 years | 53.8 |  | Ref | 1 | - | - |  | Ref | 1 | - | - |
|  | 40–49 years | 63.3 |  | 0.161 | 1.18 | 1.14–1.21 | < 0.001 |  | 0.181 | 1.20 | 1.16–1.23 | < 0.001 |
|  | 50–64 years | 69.5 |  | 0.255 | 1.29 | 1.26–1.32 | < 0.001 |  | 0.277 | 1.32 | 1.28–1.35 | < 0.001 |
|  | 65–74 years | 75.7 |  | 0.341 | 1.41 | 1.37–1.44 | < 0.001 |  | 0.357 | 1.43 | 1.39–1.47 | < 0.001 |
|  | 75–83 years | 79.3 |  | 0.388 | 1.47 | 1.43–1.52 | < 0.001 |  | 0.406 | 1.50 | 1.45–1.55 | < 0.001 |
| Sex | | | | | | | | | | | | |
|  | Women | 69.7 |  | Ref | 1 | - | - |  | Ref | 1 | - | - |
|  | Men | 59.2 |  | -0.163 | 0.85 | 0.84–0.86 | < 0.001 |  | -0.122 | 0.89 | 0.87–0.90 | < 0.001 |
| COVID-19 vaccination | | | | | | | | | | | | |
|  | 3 times or more | 68.4 |  | Ref | 1 | - | - |  | Ref | 1 | - | - |
|  | 1 or 2 time(s) | 54.8 |  | -0.220 | 0.80 | 0.78–0.83 | < 0.001 |  | -0.133 | 0.88 | 0.85–0.90 | < 0.001 |
|  | None | 51.6 |  | -0.281 | 0.76 | 0.73–0.78 | < 0.001 |  | -0.245 | 0.78 | 0.76–0.81 | < 0.001 |
| History of COVID-19 | | | | | | | | | | | | |
|  | Yes | 58.3 |  | Ref | 1 | - | - |  | Ref | 1 | - | - |
|  | No | 67.3 |  | 0.143 | 1.15 | 1.13–1.18 | < 0.001 |  | 0.058 | 1.06 | 1.04–1.08 | < 0.001 |
| Body mass index | | | | | | | | | | | | |
|  | < 18.5 kg/m^2^ | 64.1 |  | 0.041 | 1.04 | 1.02–1.07 | 0.001 |  | 0.039 | 1.04 | 1.02–1.07 | 0.002 |
|  | 18.5–24.9 kg/m^2^ | 66.8 |  | Ref | 1 | - | - |  | Ref | 1 | - | - |
|  | 25.0–29.9 kg/m^2^ | 64.8 |  | 0.012 | 1.01 | 0.99–1.04 | 0.367 |  | 0.006 | 1.01 | 0.98–1.03 | 0.629 |
|  | ≥ 30.0 kg/m^2^ | 62.7 |  | -0.022 | 0.98 | 0.93–1.03 | 0.391 |  | -0.006 | 0.99 | 0.95–1.05 | 0.818 |
| Smoking | | | | | | | | | | | | |
|  | Never | 64.7 |  | Ref | 1 | - | - |  | Ref | 1 | - | - |
|  | Past | 65.2 |  | 0.008 | 1.01 | 0.99–1.03 | 0.445 |  | -0.021 | 0.98 | 0.96–1.00 | 0.069 |
|  | Current | 61.8 |  | -0.045 | 0.96 | 0.93–0.98 | 0.003 |  | -0.041 | 0.96 | 0.93–0.99 | 0.007 |
| Comorbid conditions | | | | | | | | | | | | |
|  | No conditions | 62.5 |  | Ref | 1 | - | - |  | Ref | 1 | - | - |
|  | 1 condition | 72.3 |  | 0.146 | 1.16 | 1.13–1.18 | 0.012 |  | 0.036 | 1.04 | 1.01–1.06 | 0.002 |
|  | 2 conditions | 71.8 |  | 0.138 | 1.15 | 1.11–1.19 | < 0.001 |  | 0.037 | 1.04 | 1.00–1.08 | 0.043 |
|  | ≥ 3 conditions | 67.4 |  | 0.075 | 1.08 | 1.02–1.14 | 0.012 |  | 0.105 | 1.11 | 1.05–1.18 | < 0.001 |

RR: relative risk; CI: confidence interval; β: standardized coefficient.

*Adjusted for age, sex, annual household income, education, occupation, marital status, body mass index, and smoking.
